# Tumor sequencing before and after neoadjuvant chemoradiotherapy in locally advanced rectal cancer: Genetic tumor characterization and clinical outcome

**DOI:** 10.1016/j.ctro.2024.100894

**Published:** 2024-11-22

**Authors:** Kerstin Clasen, Nadja Ballin, Leon Schütz, Irina Bonzheim, Olga Kelemen, Michael Orth, Cihan Gani, Olaf Rieß, Stephan Ossowski, Maximilian Niyazi, Christopher Schroeder

**Affiliations:** a Department of Radiation Oncology, University Hospital Tübingen, Tübingen, Germany; b Institute of Medical Genetics and Applied Genomics, Medical Faculty and University Hospital, Tübingen, Germany; c Institute of Pathology and Neuropathology, Comprehensive Cancer Center and University Hospital Tübingen, Tübingen, Germany

**Keywords:** Radiotherapy, Next-generation sequencing, Tumor mutational burden, KRAS, TP53, ATM

## Abstract

**Background and purpose:**

Neoadjuvant chemoradiotherapy (NCRT) is a standard treatment option for locally advanced rectal cancer. However, there is still conflicting data about the genetic landscape and potential dynamics during and after NCRT. This study evaluated oncogenic driver mutations before NCRT and investigated corresponding resection samples after treatment.

**Materials and methods:**

In 17 patients the pre-therapeutic biopsy and in ten cases the related resection specimen were investigated by next-generation sequencing using a dedicated cancer panel (708 genes). Oncogenic driver mutations and tumor mutational burden (TMB) were compared pre- and post NCRT to evaluate stability of the genomic landscape. TMB and frequently detected driver mutations were correlated with outcome parameters.

**Results:**

In our corresponding tumor samples before and after NCRT 95.2 % of the oncogenic driver mutations could be found in both specimens whereas one *ATM* and one *RYR1* mutation were not detectable after NCRT. TMB decreased in all patients after neoadjuvant treatment. *KRAS* ± *TP53* mutations and TMB ≥ 5 were associated with impaired outcome.

**Conclusion:**

Most oncogenic driver mutations investigated persisted after neoadjuvant treatment. At the same time, we did not observe ascending TMB after treatment but decline. Thus, NCRT does not seem to induce a relevant number of new driver mutations or mutational burden. Genetic profiling implies the potential to support tumor-informed approaches and outcome estimation in future.

## Highlights

• Most oncogenic driver mutations could be detected before and after NCRT.
• Solely one *ATM* and one *RYR1* mutation were not detectable after NCRT.
• The tumor mutational burden (TMB) decreased after treatment.
• *ATM* might account for radiosensitive (sub-)clones.
• Higher TMB ≥ 5 and *KRAS* ±  *TP53* mutations were correlated with impaired outcome.

## Introduction

A multimodality approach including neoadjuvant chemoradiotherapy (NCRT) has been a standard treatment strategy in locally advanced rectal cancer patients since many years [1], [2], [3]. After initial biopsy and diagnosis, radiotherapy of five to six weeks with concomitant chemotherapy is followed by surgical resection. Yet, a relevant number of patients achieves a pathologic complete response (pCR) after NCRT [4], [5] and could qualify for organ preservation strategies in terms of a “watch and wait” management. In others, relevant tumor mass can still be found in the resection specimen and intensified therapeutic approaches might be reasonable. In recent therapeutic concepts like total neoadjuvant therapy (TNT), organ preservation strategies and dose escalation trials, NCRT is still an integral part of current study designs [6], [7], [8].

For patient stratification and personalized treatment approaches, genetic testing and insights into tumor evolution are desirable. Furthermore, genetic biomarkers like cell-free tumor DNA (ctDNA) are under investigation. In a recent report, we observed different courses of ctDNA during NCRT in this rectal cancer cohort which were correlated with imaging response [9].

In patients who received tumor resection, two pathologic specimens are theoretically available for biomarker investigations: pre- and post-neoadjuvant treatment. However, data about potential genetic changes or evolution due to neoadjuvant treatment is limited and conflicting [10], [11], [12], [13], [14], [15].

In rectal cancer, the predictive and prognostic value of recurrently detected genetic driver mutations like *APC*, *KRAS* and *TP53* is still under debate. However, a negative impact of *KRAS* and *KRAS* / *TP53* co-occurrence on tumor response and outcome has been suggested previously [10], [16].

Aim of our prospective biomarker study was to compare sequencing results of tumor samples before and after NCRT. We intended to evaluate the genomic landscape of these tumors and to investigate if particular driver variants disappear or even newly arise during NCRT. Furthermore, we correlated outcome with frequently detected driver variants and genomic features.

## Materials and methods

### Patients and treatment

This pilot biomarker study recruited patients with locally advanced rectal cancer between 2016 and 2017. Beforehand, the study hast been approved by the local ethics committee (734/2015BO2) and all procedures were performed in compliance with relevant laws and institutional guidelines. Twenty patients were initially included and declared their written informed consent, however, one patient dropped out due to a cardiac event before treatment start. Two further patients were excluded due to technical issues and lack of tumor content in the respective specimens. Thus, 17 patients could be included in this study.

After diagnosis by endoscopic biopsy and staging to rule out metastatic disease, all patients received neoadjuvant long-term chemoradiotherapy up to 50.4 Gy in 28 fractions with two concurrent cycles of 5-Fluorouracil (5-FU). Additionally, deep regional hyperthermia was applied in six patients (twice weekly, mean: 8 sessions, range: 2–10 sessions). After surgical tumor resection (mean after 7 weeks, range 4–11 weeks), adjuvant chemotherapy with 5-FU was administered in the majority of patients.

### Genetic sampling and tissue analyses

For genetic biomarker evaluation, tumor samples (formalin-fixed paraffin-embedded tissue, FFPE) of the biopsy and the resection specimen as well as corresponding normal tissue (adjacent tumor-free tissue) were provided from the pathology department. All coding genomic target regions (708 genes), the adjacent intron areas as well as selected individual introns (necessary for the detection of certain fusions) were enriched with the in-solution technology from Agilent (SureSelectXT, Agilent, Santa Clara, USA). Indexed samples were finally pooled and sequenced to approximately 700× (tumor) or 300× (normal) depth on a NovaSeq 6000 instrument (Illumina, San Diego, USA) using 2 × 100 Paired-End sequencing protocol.

The raw data were demultiplexed using Illumina bcl2fastq and converted into FASTQ files. The data was processed using the in-house bioinformatics pipeline megSAP (https://github.com/imgag/megSAP). In short, reads were mapped against the reference genome using bwa-mem (https://github.com/lh3/bwa), variants were called with strelka2 [17] and annotated with VEP [18]. Tumor mutational burden (TMB) was calculated with the number of variants in the coding region of the analyzed gene panel normalized for the panel size. The TMB was corrected for the effect of variants in tumor suppressor genes which are likely to be overrepresented in panel sequencing. The comparison values listed were published for different tumor entities [19]. To analyze the impact of TMB on outcome parameters, we used the cutoff of 5 to group patients in accordance with previous reports [20]. MANTIS was used to detect changes in the sequence length of microsatellites [21]. A metric was obtained that estimates the extent of changes in the microsatellites. We used a value of 0.16 to differentiate between microsatellite-stable and microsatellite-unstable tumors.

Potentially clinically relevant somatic changes were prioritized and evaluated with the in-house software GSvar (https://github.com/imgag/ngs-bits). Somatic variant classification was performed according to ClinGen/CGC/VICC SOP for the classification of pathogenicity of somatic variants in cancer (oncogenicity) version 1.0 [22].

For patients 104, 105 and 108 the driver mutations that were found in the initial sample were evaluated manually due to low tumor content in the resection sample. In patient 113 (low coverage of the biopsy sample) standard filters were relaxed and hits were verified manually. For both driver mutations that we only detected pre-therapeutically (i.e. *ATM* in patient 101 and *RYR1* in patient 108), the respective sequences were checked manually for these variants in the raw data of the resection sample to confirm the results.

Cross-contaminations with DNA material from an unrelated tumor sample were detected in NGS panel sequencing of the resection specimen of patient 101 and the tumor biopsy of patient 109. Tumor-specific alterations of the contaminant were present at low allele frequency (Pat. 101: 9 %, Pat. 109: 13 %), which impaired correct calculation of TMB. However, analyses of somatic mutations in the studied tumor samples was not affected.

### Outcome analysis and statistics

To evaluate pathologic response, we used the Dworak regression scale. To correlate well-known driver mutations (i.e. *APC*, *KRAS* and *TP53*) with outcome parameters we used all sequenced tumor specimens. If in one specimen (pre- and/or post-therapy) an oncogenic driver variant was found, we considered this variant as proven in this patient. For statistics, we used IBM SPSS version 28. For group comparisons Mann-Whitney U-Test and Wilcoxon signed rank test were used. Furthermore, metastasis-free survival and overall survival were estimated by the Kaplan-Meier method and the log-rank test. P-values <0.05 were considered significant. Values between 0.05 and 0.1 were reported as trends.

## Results

Seventeen patients could be included in this analysis. Patients’ characteristics are shown in Table 1. Five patients developed metastatic disease during follow-up whereas no patient showed local failure. All patients presented with pre-therapeutic cT3 tumors. We tested if the nodal stages (cN0, cN1, cN2) had an effect on outcome in our cohort but did not find significant differences (data not shown). We did not find indications of microsatellite instability (MSI) in any of our included patients (data not shown). Thus, our oncogenic driver analysis comprehends a homogenous group of microsatellite-stable (MSS) tumors.

**Table 1:** Patient characteristics of 17 patients whose tumors could successfully be sequenced and evaluated.

***Age (years, range)***		
Mean	63	37–79

**Gender *(n, %)***		
Female	3	17.7 %
Male	14	82.3 %

***Clinical staging (n, %)***		
cT3 cN0	2	11.8 %
cT3 cN1	7	41.2 %
cT3 cN2	8	47.0 %
cT4 any cN	0	0 %

**Dworak regression grade *(n, %)***		
1 (minimal response)	5	29.4 %
2 (moderate response)	6	35.3 %
3 (near-complete response)	4	23.5 %
4 (complete response)	2	11.8 %

**Interval RT − Resection *(days, range)***		
Mean	51	30–76

**Localization *(n, %)***		
0–5 cm from anal verge	3	17.7 %
5–10 cm from anal verge	10	58.8 %
>10 cm from anal verge	4	23.5 %

Abbreviations: CRT = Chemoradiotherapy.

In ten patients we could sequence tumor samples before and after NCRT and compare the respective results. In six patients, only the pre-therapeutic biopsy was evaluable due to pCR (n = 2) and lack of tumor tissue in the provided FFPE specimen from surgery (n = 4). In patient 106 we excluded the initial biopsy due to low coverage and only evaluated the resection specimen in this study.

**Fig. 1:**
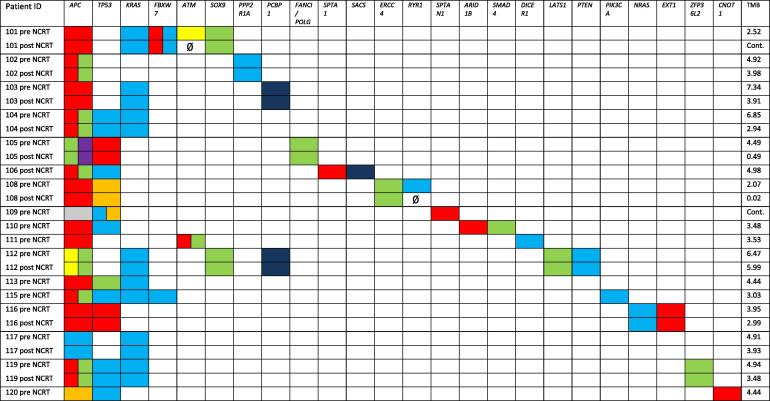
Heatmap of the oncogenic driver variants found in the pre- and post-neoadjuvant chemoradiotherapy (NCRT) specimens. Most mutations were found in both, the pre- and the post NCRT samples (where applicable). If variants were only detected in one sample, the paired field was indicated by “Ø”. Colors are encoding the variant types: stop – red; missense – light blue; frameshift – light green; splice donor – orange; splice acceptor – yellow; splice region – grey; multihit – dark blue; deletion and insertion – purple. Two samples were likely contaminated (Cont.), so we excluded them from tumor mutational burden (TMB) analysis. (For interpretation of the references to colour in this figure legend, the reader is referred to the web version of this article.).

In the 17 patients evaluable for variant analysis, 71 oncogenic driver mutations could be detected in total. We found several well-known oncogenic driver mutations like *APC* (n = 17; 100 %), *TP53* (n = 11; 65 %), *KRAS* (n = 8; 47 %) and *FBXW7*, *ATM*, *PCBP1* and *SOX9* (n = 2; 12 % respectively). A comprehensive overview of all detected drivers and the respective pre- and post-treatment samples as well as corresponding TMB are shown in Fig. 1. We could detect 42 oncogenic driver mutations in the paired samples (n = 10 pairs). Of these, 40 (95.2 %) drivers could be found in both associated samples of the same patients, whereas two (4.8 %) mutations were found only in the pre-therapeutic sample (i.e. *ATM* in patient 101 and *RYR1* in patient 108 (with subclonal allele frequency)). Thus, in respect of oncogenic driver variants, we found broad stability of the genomic landscape in our corresponding samples before and after NCRT.

We tested for mutational signatures of pre- and post-chemoradiation samples but did not find any 5-FU or radiation associated signatures (data not shown).

**Fig. 2:**
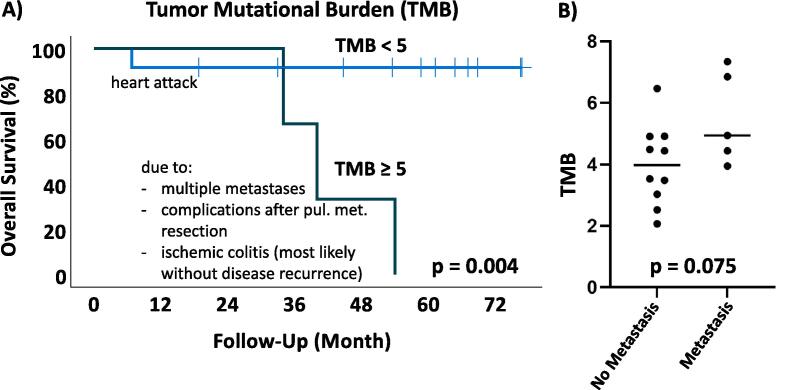
Overall survival was impaired by the pre-therapeutic incidence of tumor mutational burden (TMB) ≥ 5 (A). The causes of death are added to the events in the Kaplan-Meier curves. Higher initial TMB showed a trend to occurrence of metastatic disease (B).

Nine paired samples could be evaluated for TMB analysis as two samples had to be excluded due to assumed contamination (one of them was part of a “paired sample”). The highest TMB in our cohort was 7.34. Thus, no patient showed hypermutation (TMB > 20). TMB showed a decrease after NCRT in 100 % of the evaluable corresponding samples (p = 0.008). Lower pre-therapeutic TMB (<5) was significantly associated with improved overall survival (n = 15; p = 0.004; Fig. 2a). Furthermore, a trend to significance could be seen when correlating higher initial TMB (≥5) and the occurrence of metastatic disease during follow-up (n = 15; p = 0.075; Fig. 2b).

When correlating the most recurrently detected oncogenic driver variants (*TP53*, *KRAS*) with outcome parameters, no significant correlation with pathologic response could be found (data not shown). In our cohort, all patients presented with an oncogenic driver mutation in *APC*. Thus, the impact of a co-occurrent *APC* mutation in our cohort is not evaluable.

**Fig. 3:**
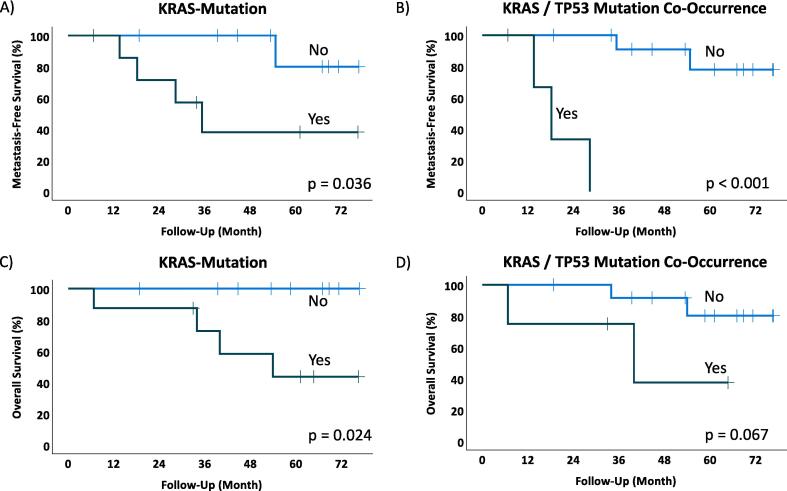
Driver mutations and outcome. Patients with mutations in *KRAS* (A) and *KRAS*/*TP53* co-occurrence (B) showed inferior metastasis-free survival. Furthermore, *KRAS* mutations were associated with impaired overall survival (C) and a trend to worse overall survival could be found if both, *KRAS* and *TP53* were mutated (D).

In patients with *KRAS* or co-occurrent *KRAS* / *TP53* mutations, a significant negative impact on metastasis-free survival was found (p = 0.036/p < 0.001; Fig. 3a and b). Overall survival was significantly impaired in patients having a *KRAS* mutation and a trend could be observed in case of co-occurrent *KRAS* / *TP53* mutations (p = 0.024/p = 0.067; Fig. 3c and d).

## Discussion

The spectrum of the oncogenic driver mutations we found in our cohort is well in line with previous reports of The Cancer Genome Atlas Network (TCGA, 2012) [23] and a recent study of Chatila et al. (2022) that analyzed 692 genomic profiles of treatment-naive patients with rectal cancer [24]. Most frequently mutated genes in our microsatellite stable cohort were APC (100 %; TCGA 81 %; Chatila et al. 81 %), TP53 (65 %; TCGA 60 %; Chatila et al. 81 %) and KRAS (47 %; TCGA 43 %; Chatila et al. 42 %). Slightly divergent percentages are most likely explained by our comparably small cohort. Thus, our cohort shows representative genetic features for further investigations.

In comparison of related samples before and after neoadjuvant treatment, the vast majority of driver mutations could be detected in both samples, respectively. Thus, NRCT does not seem to induce relevant numbers of newly arising driver mutations or broadly select for subclonal tumor fractions that were not detectable pre-therapeutically. These findings might be helpful if target therapy approaches and re-biopsies are discussed after radiation treatment. Genetic concordance of findings before and after NCRT is well in line with two studies from the US and Ireland [10], [11].

Kamran et al. reported sequencing results of 17 patients with rectal cancer (whole exome sequencing (WES), sequencing depth on average 150 X) [10]. In 14 patients pre- and post-neoadjuvant chemoradiotherapy specimens could be analyzed (3 × PCR) and no case was associated with MSI. No impact of neoadjuvant therapy on global somatic mutations could be found and TMB did not change significantly. In accordance with these results, a previous study in cooperation of the Beaumont Hospital in Dublin and the MD Anderson Cancer Center in Texas investigated 12 pre- and post-chemoradiotherapy samples and did not find relevant genomic tumor evolution in response to neoadjuvant treatment (WES mean on target coverage 83 X /34 X; additional Panel 368 X />5000 X) [11]. Solely one post-therapy sample revealed a *TP53* variant that was not detected earlier and a new *HRAS* mutation was detected in another patient. TMB did not show significant difference between the pre- and post-treatment specimens in this study. However, in one patient, a new mutation of *MSH2* was seen during therapy and in another patient, an enhanced allele frequency of *MSH2* (accompanied by elevated mutational burden) was detected in the post-treatment sample. In contrast to the study of Toomey et al. we did not observe any development or selection of microsatellite instability in the resection specimens due to chemoradiotherapy [11].

In opposition to these studies suggesting stability of the genomic landscape after NCRT, two studies from Korea [12] (n = 11 pairs of pre- and post-treatment samples, WES, sequencing depth in average: 30X) and China [13] (n = 28 pairs of pre- and post-treatment samples, WES with 98X, in 22 selected samples target region sequencing of 78 genes >1,000X, only 8 % shared mutations) reported relevant changes in genomic features after neoadjuvant chemoradiation.

This reported discrepancy of genetic persistence versus genetic alterations in response to neoadjuvant treatment might partly be due to methodology differences in regard to whole exome approaches or panel sequencing with divergent sequencing depths. Furthermore, in the Korean cohort, several patients with mutations in genes that are associated with MSI were included which might influence response to chemoradiation, as well. In our study no patient showed indications of MSI. Furthermore, confounding regional or ethnic differences between the cohorts cannot be ruled out.

Tumors with *ATM* mutations are of interest in radiotherapy as *ATM* is involved in the repair of radiation-induced DNA double-strand breaks [25]. A previous report supports the idea of radiosensitive phenotypes of tumors with somatic *ATM* inactivation [26]. Interestingly, we found *ATM* driver mutations in patient 101 and patient 111. This mutation was not found in the resection specimen of patient 101 whilst all other drivers could be detected in both specimens in this patient. Patient 111 showed pCR in the resection specimen. In both cases, one could speculate about radiosensitive clones due to *ATM* mutations that disappeared during NCRT. However, this data has to be considered as solely descriptive as numbers are too small for conclusions. Further investigations are required to explore the role of *ATM* mutations in radiotherapy.

We did not find rising TMB in response to neoadjuvant chemoradiation which is in line with previous reports [10], [11]. In contrast, even though most oncogenic driver mutations persisted after NCRT, TMB decreased in all paired samples. Even though our data must be handled with care due to low tumor content in some resection samples and the small cohort, potentially arising treatment-related new variants (e.g. by radiation-induced DNA damage) do not seem to be a relevant issue. This finding supports radio-oncologic approaches in rectal cancer in future.

The prognostic potential of TMB was investigated for immunotherapy-naive patients before [20]. A parabolic correlation of TMB and outcome was reported as low TMB (<5) and (very) high TMB (≥20/≥50) were correlated with fortunate overall-survival whilst intermediate TMB (≥5 < 20) showed decreased survival. The authors hypothesize, that the dismal outcome of patients with intermediate TMB compared to low TMB might be explained by higher numbers of drivers and the “mutator phenotype” [27] whilst, after reaching a peak, the innate immune response on (very) high TMB supports the positive prognostic effect in these patients [20]. With all the caveats of our small cohort we did observe a trend to dismal metastasis-free survival and significantly inferior overall survival in patients with TMB ≥ 5 which is in accordance with these previous results. Alike, Lee et al. found a correlation of higher pre-therapeutic TMB and dismal outcome [12]. Therefore, also in patients who do not receive immunotherapy, TMB seems to be a potential biomarker for outcome estimations and personalized treatment strategies.

We investigated if specific recurrently detected mutations (i.e. *KRAS* and *TP53* and their co-occurrent presence) influence response and oncologic outcome. A negative impact of *KRAS* mutations and combined *KRAS* / *TP53* mutations was reported regarding non-PCR and resistance to neoadjuvant chemoradiation [16], [28]. Furthermore, *KRAS* [24] and *TP53* [29] seem to impair PFS. Kamran et al. found concurrent *KRAS*/*TP53* mutations to be associated with worse pathologic response and dismal PFS and suggested this genotype to support local immune escape [10]. In line with these results, we found impaired metastasis-free survival and overall survival for patients with *KRAS* mutations as well as worse metastasis-free survival for co-occurrent *KRAS*/*TP53* mutations. These genetic profiles might qualify for personalized medicine approaches and inform risk stratification in organ preservation strategies in future.

Our study is limited by several criteria. First, our cohort is limited in numbers, so our results have to be considered as pilot data and preliminary due to the small sample size and needs to be validated in bigger studies. Second, we used a panel approach for tumor-normal sequencing instead of whole exome or whole genome sequencing to enhance target coverage. Even though a large number of onco-drivers was tested, we might have missed some driver mutations by this approach and technical limitations to calculate TMB and signatures have to be considered. Furthermore, FFPE samples after NCRT contained little tumor content in several cases which might cause inaccuracies of genetic findings and underestimation of TMB.

## Conclusion

Overall, this pilot biomarker study revealed several interesting findings about corresponding tumor samples before and after NCRT regarding stability of the genetic landscape. Furthermore, prognostic genetic phenotypes might support personalized radiotherapeutic approaches in future.

## Ethics approval and consent to participate

All patients declared their (written) informed consent to participate and this study was approved by the local ethics committee in 2015 (reference number 734/2015BO2). The study was performed in accordance with the ethical standards as laid down in the 1964 Declaration of Helsinki and its later amendments.

## Consent for publication

All patients declared their consent to publish the data of the study.

## CRediT authorship contribution statement

**K. Clasen:** Conceptualization, Project administration, Investigation, Formal analysis, Writing – original draft, Visualization, Funding acquisition. **N. Ballin:** Investigation, Formal analysis, Data curation. **L. Schütz:** Software, Formal analysis, Data curation. **I. Bonzheim:** Resources, Writing – review & editing. **O. Kelemen:** Investigation, Writing – review & editing. **M. Orth:** Writing – review & editing, Supervision. **C. Gani:** Conceptualization, Validation, Supervision. **O. Rieß:** Resources, Supervision. **S. Ossowski:** Software, Resources, Supervision. **M. Niyazi:** Resources, Writing – review & editing, Supervision. **C. Schroeder:** Conceptualization, Investigation, Formal analysis, Data curation, Writing – original draft.

## Funding

KC has been supported by intramural research funds (Fortüne/PATE) of the University Hospital and the Faculty of Medicine, Eberhard-Karls-University of Tübingen (Funding numbers 2600-0-0 and 2447-0-0). Furthermore, KC received funding from the Research Seed Capital fund (RiSC) 2020 of the Ministry of Science, Research and Arts Baden-Württemberg. SO and CS are supported by funding from the European Union’s EU4Health programme under grant agreement No 101080009 and the DKTK EXLIQUID consortium. No sponsor was involved in the study design, the collection, analysis and interpretation of data, or in the writing of the report. We acknowledge support from the Open Access Publication Fund of the University of Tübingen.

## Declaration of competing interest

The authors declare the following financial interests/personal relationships which may be considered as potential competing interests: KC, CG and MN report institutional collaborations including financial and non-financial support by Elekta, Philips, Siemens, Dr. Sennewald, PTW Freiburg, Kaiku and Therapanacea. CS reports institutional grants from Novartis and Illumina as well as research grants from BMS Stiftung Immunonkologie outside the submitted work.

## Data Availability

The datasets analyzed during the current study are available from the corresponding author upon reasonable request.
